# Postglacial range shift and demographic expansion of the marine intertidal snail *Batillaria attramentaria*

**DOI:** 10.1002/ece3.1374

**Published:** 2014-12-28

**Authors:** Phuong-Thao Ho, Ye-Seul Kwan, Boa Kim, Yong-Jin Won

**Affiliations:** 1 Division of EcoCreative, Ewha Womans University52 Ewhayeodae-gil, Seodaemun-gu, Seoul, 120-750, Korea; 2 Division of EcoScience, Ewha Womans University52 Ewhayeodae-gil, Seodaemun-gu, Seoul, 120-750, Korea; 3 Department of Life Science, Ewha Womans University52 Ewhayeodae-gil, Seodaemun-gu, Seoul, 120-750, Korea

**Keywords:** *Batillaria attramentaria*, Bayesian skyline plot, climate change, *COI* gene, demographic expansion, range shift, the last glacial maximum

## Abstract

To address the impacts of past climate changes, particularly since the last glacial period, on the history of the distribution and demography of marine species, we investigated the evolutionary and demographic responses of the intertidal batillariid gastropod, *Batillaria attramentaria*, to these changes, using the snail as a model species in the northwest Pacific. We applied phylogeographic and divergence population genetic approaches to mitochondrial *COI* sequences from *B. attramentaria*. To cover much of its distributional range, 197 individuals collected throughout Korea and 507 publically available sequences (mostly from Japan) were used. Finally, a Bayesian skyline plot (BSP) method was applied to reconstruct the demographic history of this species. We found four differentiated geographic groups around Korea, confirming the presence of two distinct, geographically subdivided haplogroups on the Japanese coastlines along the bifurcated routes of the warm Tsushima and Kuroshio Currents. These two haplogroups were estimated to have begun to split approximately 400,000 years ago. Population divergence analysis supported the hypothesis that the Yellow Sea was populated by a northward range expansion of a small fraction of founders that split from a southern ancestral population since the last glacial maximum (LGM: 26,000–19,000 years ago), when the southern area became re-submerged. BSP analyses on six geographically and genetically defined groups in Korea and Japan consistently demonstrated that each group has exponentially increased approximately since the LGM. This study resolved the phylogeography of *B. attramentaria* as a series of events connected over space and time; while paleoceanographic conditions determining the connectivity of neighboring seas in East Asia are responsible for the vicariance of this species, the postglacial sea-level rise and warming temperatures have played a crucial role in rapid range shifts and broad demographic expansions of its populations.

## Introduction

From a paleoceanographic perspective, the seas surrounding the Korean peninsula, including the Yellow Sea, the East Sea/the Sea of Japan, the South Sea of Korea, and the East China Sea, have been profoundly affected by climate and sea-level changes during the Quaternary period. In particular, during the last glacial maximum (LGM) approximately 26,000–19,000 years ago (26–19 ka) (Clark et al. 2009), the sea level was approximately 130 m lower and the air temperature was approximately 8–13°C lower relative to present levels (Park and Yi 1995). Due to the characteristics of the continental shelf of East Asia, excessively widespread shallow seas such as the East China Sea (ECS) and the Yellow Sea (YS), a great portion of the shelf that had been submerged during the interglacial periods was then exposed to air by marine regression. As a result, the coastlines in this region are generally complex and have also substantially changed, with a repeating pattern of regression and transgression across wide ranges following glaciation/interglaciation cycles that generate or cover land bridges (Kim et al. 2000; Ryu et al. 2008).

Consequently, tremendous fluctuations in sea level and temperature accompanying connections or disconnections of lands became to draw scientific attention to their role in biogeographic changes in both terrestrial and marine taxa during the Quaternary in East Asia (Amano 2004; Kiyoshi and Sota 2006; Zhang and Sota 2007; Sekin et al. 2013). Apart from these local events, the question about the impacts of past climate and sea-level changes on the distribution and evolution of marine organisms was an important theme of marine evolutionary biology and began to be answered by phylogeographic analyses with molecular genetic data (Hellberg et al. 2001). Over the last decade, there were a growing number of studies showing the climatic effects on the demography and biogeography of marine and terrestrial species. For instance, demographic expansions in response to past climate changes have been observed in numerous marine (e.g., Table1, Grant et al. 2012) and terrestrial species worldwide (e.g., Timothy and Frank 2008; Liao et al. 2010; Camargo et al. 2013), suggesting that population growth is a characteristic effect of climate changes during the Late Pleistocene.

**Table 1:** Genetic diversity in Korean *Batillaria attramentaria* populations

Group and localities	Sample size	Haplotype diversity (*h*)	Nucleotide diversity (*π*)	Average number of pairwise differences (*k*)
Yellow Sea (YS)	72	0.302 ± 0.069	0.016 ± 0.017	0.375 ± 0.362
Dobido	16	0.350 ± 0.148	0.024 ± 0.023	0.575 ± 0.490
Gonam	30	0.131 ± 0.082	0.006 ± 0.009	0.133 ± 0.204
Byunsan	11	0.000 ± 0.000	0.000 ± 0.000	0.000 ± 0.000
Jindo	15	0.591 ± 0.077	0.027 ± 0.025	0.648 ± 0.531
South Sea (SS)	39	0.405 ± 0.096	0.025 ± 0.022	0.591 ± 0.484
Yeosu	15	0.448 ± 0.135	0.020 ± 0.020	0.476 ± 0.437
Geoje	13	0.295 ± 0.156	0.013 ± 0.016	0.308 ± 0.338
Masan	11	0.436 ± 0.133	0.036 ± 0.031	0.873 ± 0.661
North of Jeju Island (NJI)	56	0.204 ± 0.072	0.020 ± 0.020	0.491 ± 0.428
Shinchang	12	0.000 ± 0.000	0.000 ± 0.000	0.000 ± 0.000
Hyubje	17	0.228 ± 0.130	0.010 ± 0.013	0.235 ± 0.286
Hanlim	12	0.167 ± 0.134	0.007 ± 0.011	0.167 ± 0.240
Samyang	8	0.250 ± 0.180	0.010 ± 0.015	0.250 ± 0.311
Sewha	7	0.476 ± 0.171	0.099 ± 0.070	2.381 ± 1.468
South of Jeju Island (SJI)	30	0.616 ± 0.074	0.096 ± 0.060	2.301 ± 1.297
Shinyang	17	0.559 ± 0.083	0.025 ± 0.023	0.603 ± 0.504
Kangjeong	6	0.867 ± 0.129	0.161 ± 0.109	3.867 ± 2.256
Pyosun	7	0.476 ± 0.171	0.198 ± 0.126	4.762 ± 2.647

Although there is still a lack of phylogeographic studies on the issue of historical demography of marine taxa in East Asia, several examples could be found from marine fishes and mollusks. Evident are genetic signals of historical demographic growths in fish species (Liu et al. 2006; Han et al. 2008; Kwan et al. 2012) and mollusks including the bivalve pen shell *Atrina pectinata* (Xue et al. 2014) and gastropod species such as the moon turban snail *Lunella granulata* (Chiu et al. 2013), the Japanese turban shell *Turbo cornutus* (Kojima et al. 1997, 2000), and the intertidal snail *Batillaria attramentaria* (Kojima et al. 2004). In addition to the evidence of population expansions, all the latter gastropod species commonly showed that the current population genetic patterns of them have been primarily shaped by the influence of regional geographic barriers generated by lands and divergent oceanic currents. The existence of distinct genetic groups largely corresponding to different regimes of ocean currents was commonly found in the three gastropod species although they have different modes of larval development. Therefore, these findings really tell us that the understanding of historical changes of marine taxa in their distribution and demography in East Asia should be viewed or tested under the light of past climate changes and regional ocean currents as well.

Methodologically, most of these previous studies were based on a prespecified parametric model of demographic history, such as exponential growth (Rogers and Harpending 1992). In reality, however, demographic histories of natural populations are more complex than those inferred by the exponential model. Therefore, Bayesian skyline plot (BSP) methods, which infer changes in population size over time from a sample of nucleotide sequences on the basis of coalescent theory (Pybus and Rambaut 2002; Drummond et al. 2005), were developed to facilitate a more realistic understanding of complex demographic history. A reconstructed demographic history of a species by these methods, in turn, provides insights into the question how the species responded to past climate and habitat changes. For instance, the demographic histories of the bowhead whale in Arctic (Liu et al. 2006; Foote et al. 2013) and the sand goby in the northeastern Atlantic (Larmuseau et al. 2009) could be traced to the Late Pleistocene. The bowhead whale showed a highly resolved exponential demographic expansion at approximately 10,000 years before present, contrasting to growth patterns in lineage-specific groups of the sand goby.

The intertidal snail, *Batillaria attramentaria* (Sowerby, 1855), is widely distributed along the entire coastlines of Japan, Korea, and eastern China. As described above, this region encompasses highly complex oceanographic features such as numerous islands, widespread shallow seas, and narrow and shallow straits, which have significantly amplified the effects of climate changes during the Quaternary period, particularly on the position of coastlines. As the habitats of *B. attramentaria* are confined to narrow intertidal zones with bottoms of rocks, boulders, and sandy mud along coastlines (Adachi and Wada 1997), past changes to coastlines driven by the cycle of glacial and interglacial periods would have caused distributional range shifts of *B. attramentaria* and corresponding demographic changes. In particular, the regression and transgression of the Yellow Sea following the cycle of glaciation provide an exceptional natural laboratory in which to test to what degree and when climate and paleoceanographic changes during the Quaternary period left genetic signatures of demographic changes and/or divergence in *B. attramentaria* and other marine species. In conclusion, all of these environmental and biological circumstances of *B. attramentaria* lead us to set a working hypothesis in such that this species might serve as a faithful indicator or model species for the study of the impacts of past climate changes followed by paleoceanographic changes on the evolution of gastropods in East Asia.

Previously, the phylogeography of *B. attramentaria* was investigated throughout the Japanese Islands by Kojima et al. (2004); however, most of the adjoining Korean peninsula remains to be studied. Thus, we extended the previous study by collecting new population mitochondrial *COI* sequences from Korea and pooling them with Kojima et al.'s *COI* data (2004) to obtain a more comprehensive phylogeography of *B. attramentaria* with a special focus on a detailed distributional and demographic history. To this end, we applied coalescent-based Isolation with Migration (IM) and Bayesian skyline plot (BSP) methods to obtain a more realistic and detailed history of *B. attramentaria* at the population level from *COI* sequence data.

## Materials and Methods

### Samples collection and DNA isolation

**Figure 1:**
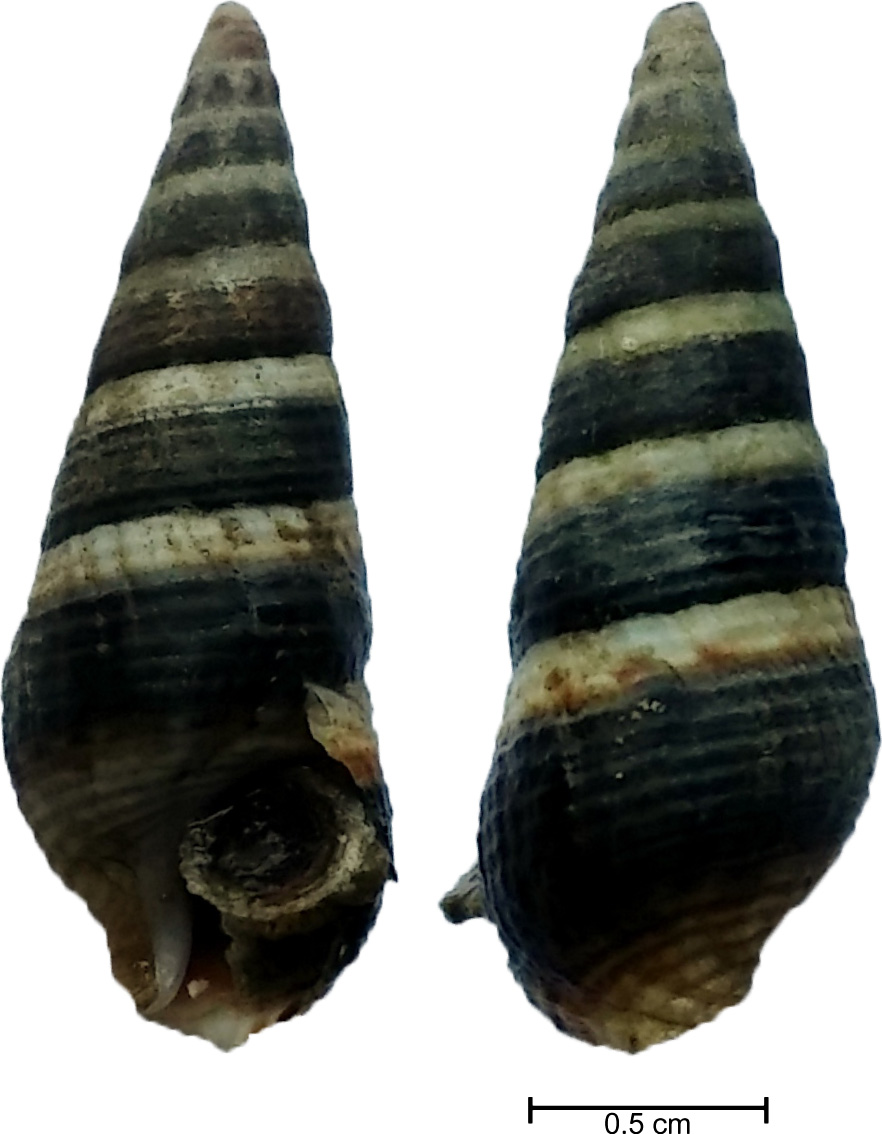
Photograph of the intertidal snail *Batillaria attramentaria* (Sowerby, 1855) collected in Korea.

**Figure 2:**
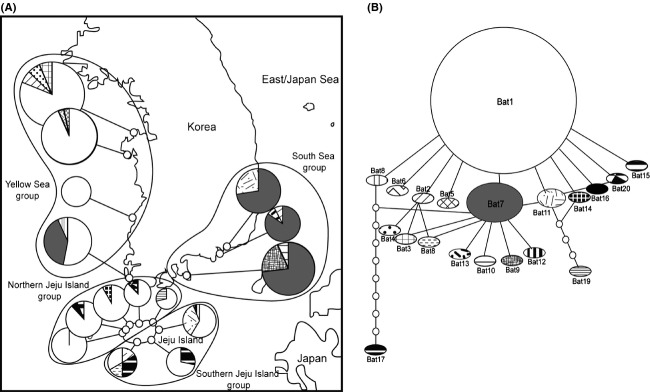
(A) Map of sampling sites for *Batillaria attramentaria* around the Korea peninsula. The pie diagrams represent frequencies of mitochondrial *COI* gene haplotypes in each population, with hatching patterns corresponding to each unique haplotype observed in this study. Boundary lines with group names illustrate how we pooled the fifteen populations into four geographically representative groups: Yellow Sea (YS), South Sea (SS), northern Jeju Island (NJI), and southern Jeju Island (SJI). (B) Minimum spanning network of twenty unique haplotypes from the fifteen *B. attramentaria* populations. Ovals represent unique haplotypes, and the size of each circle is proportional to the frequency of each haplotype. The small white circles represent missing haplotypes in our sample collections.

A total of 197 individual *Batillaria attramentaria* were collected from fifteen sites representing the widest distribution around the Korean peninsula in 2006–2007 (Table1 and Figs.1, 2). Although we tried to collect *B. attramentaria* from the east coast of the Korean peninsula, where the seafloor is mostly sandy bottom with steep gradients and no mud flats, but no live individuals were found. Upon collection, all samples were temporarily stored in 95% ethanol, delivered to our laboratory, and then preserved at −50°C prior to dissection of foot tissues for the extraction of genomic DNA. The genomic DNA was extracted from the head–foot region of each individual with the LaboPass ™ Tissue Mini Kit (Cosmogenetech Inc., Seoul, Korea), following the manufacturer's protocols.

### Polymerase chain reaction (PCR) and sequencing

The mitochondrial *COI* gene (619 bp) was amplified by polymerase chain reaction (PCR). The reaction mixture used in PCR was composed of 10X Taq polymerase buffer, 2.5 mmol/L dNTP mix, 10 *μ*mol/L of each primer, the total extracted DNA, and 2.5 units per *μ*L of Taq polymerase (all chemical substances obtained from Cosmo Genetech Inc., Seoul, Korea). PCRs were performed under the following conditions for 35 cycles: 94°C for 30 sec, 50°C for 60 sec, and 72°C for 60 sec, with a final extension step at 72°C for 7 min. Two primers were used: Bat-*COI*-4MF (5′-CAC GAC GTT GTA AAA CGA CTT AGC WGG TGT TTC CTC T-3′) and Bat-*COI*-2MR (5′-GGA TAA CAA TTT CAC ACA GGC CRA ATA ARG CRA AAA CAG C-3′). These two primers were designed with reference to the mitochondrial *COI* sequences of closely related species: *B. attramentaria*,*B. zonalis*,*B. felctosiphonata*, and *B. multiformis* (GenBank Accession: No. AB054364 to AB054367). PCR products were bidirectionally sequenced using an ABI 3730xl automatic sequencer (Applied Biosystems, Foster City, CA).

### Phylogeographic analyses of sequence data

All raw DNA sequences were aligned using AlignIR v.2.0 (LI-COR Inc., Lincoln, NE), and ambiguous basecalls and indels were manually adjusted. Then, these sequences were compared to *COI* sequences (507 specimens of 384 bp) from the Japanese population (GenBank Accession: No. AB164326 to AB164358) (Kojima et al. 2004). All statistics related to population genetic indices such as the number of mutations, parsimonious sites, and haplotypes were calculated in DnaSP v.4.0 (Rojas et al. 2003). In addition, various molecular density indices including haplotype diversity (*h*), the probability of two randomly chosen haplotypes being different (Nei 1987), nucleotide diversity (*π*) (Tajima 1983; Nei 1987), the mean number of pairwise differences (*k*), and their corresponding variances were calculated in Arlequin (Schneider et al. 2000). The population genetic structure of the Korean *B. attramentaria* was further investigated as follows. Differences in haplotype frequencies between populations were examined by the exact test of population differentiation (Raymond and Rousset 1995) in Arlequin v3.5.1.3 (Excoffier and Lischer 2010). The unbiased fixation indices of *F*_ST_ (Weir and Cockerham 1984) were estimated and tested for significance with 10,000 permutations. For hierarchical comparisons of the degree of genetic differentiation among populations, we assigned the populations to four groups according to their geographical locations, north to south and mainland to island (Table1). That is, the populations of Dobido, Gonam, Byunsan, and Jindo were assigned to the YS group; the populations of Yeosu, Geoje, and Masan were assigned to the SS group; the populations of Shinchang, Hyubje, Hanlim, Samyang, and Sewha were assigned to the NJI group; and the populations of Shinyang, Kangjeong, and Pyosun were assigned to the SJI group (Fig.2A and Table1). Tajima's *D* (Tajima 1989a,1989b) and Fu's *F*_*S*_ (Fu 1997) were used to test the assumption of neutral evolution. To confirm the result of the neutrality test, we also conducted the mismatch test based on the frequency distributions of pairwise differences between sequences (Rogers and Harpending 1992; Ray et al. 2003; Excoffier 2004). We reconstructed a genealogy of the unique haplotypes of *B. attramentaria* and compared the clades to two previously identified divergent clades, referred to “Kuroshio” and “Tsushima,” from Japan (Kojima et al. 2004). Phylogenetic trees were constructed by the neighbor-joining method in MEGA v.4.0 (Kumar et al. 2001), and the sister batillariid species, *Batillaria multiformis*, was used as an outgroup (GenBank Accession: No. AB054364) (Kojima et al. 2001). The relationships among haplotypes were also examined through the network-tree method with two separate sets of data: the Korean *B. attramentaria* dataset (619 bp) and another pooled Korea–Japan dataset (337 bp). Network trees based on the most parsimonious connections of haplotypes were constructed in TCS v.1.21 (Clement et al. 2000).

### Isolation with migration (IM) analyses

To address the intraspecific divergence of previously recognized two divergent haplotype groups, “Kuroshio” and “Tsushima” within the pooled Korea–Japan dataset, the Isolation with Migration model (IM) implemented in IMa2 (Hey 2010b) was applied to our data to estimate the six demographic parameters of the basic IM model: effective population sizes (*N*_1_, *N*_2_, *N*_A_), splitting time (*t*), and gene flow rates (*m*_1_ and *m*_2_). A 337-bp DNA segment that overlapped between the Korean and Japanese datasets was used for the IMa2 analysis in the HKY substitution model. As the mutation rate of the *COI* gene of *B. attramentaria* is not known, we used a range of nucleotide divergence rates of the *COI* genes from other marine gastropods: the genera of *Concholepas* (0.86% per nucleotide per million years) (Cardenas et al. 2009) and *Tegula* (2.4% per nucleotide per million years) (Hellberg and Vacquier 1999). IMa2 was run as follows: Monte Carlo Markov Chain (MCMC) with 10 million steps after one million burn-in steps. The generation time of *B. attramentaria* was tentatively assumed to be 1 year in this analysis because this information is not known.

In addition, the obvious marine transgression of the Yellow Sea northward after the last glacial period and its implications for range shifts and subsequent demographic processes in *B. attramentaria* were evaluated under the IM model for three populations (Hey 2010b). Given the genetic and geographic distances among the geographic groups of *B. attramentaria* in Korea and the paleoceanography of the Yellow Sea, we deduced that the YS group might have diverged from a hypothetical common ancestral population in the southern region of the Korean peninsula when the sea level fell below 100 m during the glacial maximum. Since then, interglacial periods would have accompanied northward coastal transgressions by the Yellow Sea as the sea level began to rise. The other geographic groups (SS, JI (pooled group of NJI, and SJI)) might have also descended from the ancestral population in the following order: ((YS, SS), JI). As Jeju Island first split off from the ancient coastline as it moved northward and as the Yellow Sea and the South Sea are connected along their coastlines both now and during the transgression, we set JI as a basal population. The order of divergence among more than three populations should be provided prior to running IMa2 (Hey 2010a). In this analysis, we excluded three individuals with Bat17, Kuroshio haplotype, from the SJI because the Kuroshio haplotype has a deeper coalescent root than the hypothetical ancestral population of the other individuals (*n *=* *194) of the Tsushima haplogroup in Korea. The same mutation rates described above were used under the HKY substitution model to convert the model parameters into demographic terms. The IM run was performed as follows: MCMC with 10 million steps after 100,000 burn-in steps with 10 chains under a heating scheme according to the IMa2 manual. Finally, likelihood-ratio tests were performed on the nested model of gene flow according to Hey and Nielsen (2007).

### Bayesian skyline plot (BSP) analyses

To investigate the demographic history of *B. attramentaria* populations around the Korean peninsula and the Japanese archipelago, Bayesian skyline plot (BSP) analyses were conducted in BEAST v.1.7.5 (Drummond et al. 2005). These analyses were separately performed with genetically distinct geographic groups of populations identified in this study and in the previous study (Kojima et al. 2004) as follows: four Korean groups (YS, SS, SJI, and NJI) and two Japanese groups (Tsushima and Kuroshio). As explained above, we also excluded the three individuals with the Bat17 haplotype in the SJI. The HKY+I model of nucleotide substitution was selected for all groups through the Akaike information criterion (AIC) with PartitionFinder (Lanfear et al. 2012). Coalescent genealogies of BSP were constructed with a lognormal relaxed-clock model for a total of 100 million generations, sampling every 1000 steps. As the mutation rate of *COI* gene of *B. attramentaria* is not known, this uncertainty was taken into account in our BSP analyses such that a range of mutation rates (0.86–2.4% per nucleotide per million years) was used as an a priori uniform distribution of the mutation rate of *COI*. Convergence to the stationary distribution and sufficient effective sampling sizes for each estimated parameter were checked using Tracer v.1.5 (Rambaut and Drummond 2009). After discarding 10% of samples as burn-in, historical estimates of effective population size were summarized by Tracer based on the previously constructed coalescent genealogies. Finally, as a comparative method for the test of population expansion, we examined mismatch distribution analyses by applying a sudden population expansion model (Rogers and Harpending 1992) on the same six geographic groups in Arlequin (Excoffier and Lischer 2010).

## Results

### Genetic diversity and differentiation of the Korean *Batillaria attramentaria*

Twenty unique *COI* gene haplotypes were observed in the 197 individuals of the intertidal snail *Batillaria attramentaria* from Korea (GenBank Accession: No. HQ709362-81), and these haplotypes contained 24 polymorphic sites ≥619 bp in length. Haplotype compositions of the 15 sampling sites (Fig.2A) and a network of the 20 unique haplotypes (Fig.2B) clearly showed a clinelike pattern in two dominant haplotypes (Bat1 and Bat7). Consequently, the haplotypes and their geographic distribution indicated geographic structure in four geographic groups running approximately from north to south: the Yellow Sea (YS), the South Sea of Korea (SS), northern Jeju Island (NJI), and southern Jeju Island (SJI) (Fig.2A). Among the haplotypes, the most frequent (Bat1) dominated all sampled habitats except for the SS group (Fig2A,2B). On the other hand, the SS group was dominated by a haplotype (Bat7) that showed a progressive decline to the southwest of the Korean peninsula. Notably, the southwest population, Jindo, included an approximately 50:50 mixture of the two dominant haplotypes (Bat1 and Bat7). As the result, high haplotype diversity (*h *=* *0.591) was observed in the Jindo population (Table1).

Three different genetic diversity indices (*h*,*π*, and *k*) across the populations and groups showed that the populations of YS, SS, and NJI are less diverse than those of SJI (Table1). As a group, SJI has the highest average values of the three indices (Table1). The average haplotype diversity (*h*) of all Korean samples was 0.546, and the nucleotide diversity (*π*) was 0.0017. These two indices were lower than those of Japanese *B. attramentaria* [0.849 and 0.012 (±0.007), respectively] (Kojima et al. 2004).

Pairwise *F*_ST_ values showed significant genetic differentiation among the four geographic groups (Table2). The highest *F*_ST_ values resulted from comparisons between SS and the other groups (0.432–0.617). On the other hand, the comparison between YS and NJI, which lie along a linear trajectory of the western coast of Korean peninsula, resulted in the lowest *F*_ST_ value (0.026), which is lower than that (0.155) of between the northern and southern areas of Jeju Island.

**Table 2:** Pairwise *F*_ST_ values (lower diagonal) and significance (upper diagonal at *α *= 0.05) between Korean populations of *Batillaria attramentaria*

	YS	SS	NJI	SJI
Yellow Sea (YS)		*	*	*
South Sea (SS)	0.613		*	*
North of Jeju Island (NJI)	0.026	0.617		*
South of Jeju Island (SJI)	0.200	0.432	0.155	

* *P *<* *0.05.

### Two divergent *COI* haplogroups of *B**. **attramentaria* in East Asia and its divergence time

**Figure 3:**
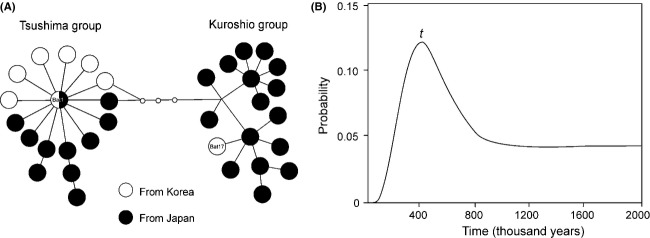
(A) Minimum spanning network of unique *COI* haplotypes reconstructed by pooling two different datasets of *Batillaria attramentaria* from Korea and Japan: overlapping 337-bp DNA sequences of *B. attramentaria* from this study (197 individuals of 619 bp) and Kojima et al. (2004) (507 individuals of 384 bp). Black and white circles represent haplotypes from Japan and Korea, respectively. Only two haplotypes, Bat1 and Bat17 (Fig.2), are marked on this network for comparison between the two different sequence datasets. Solid lines represent a single-nucleotide difference between haplotypes. (B) Posterior probability estimate for divergence time between two divergent haplogroups of Tsushima and Kuroshio collected from Korea and Japan.

The twenty unique *B. attramentaria* haplotypes found in Korea diverged into two lineages, one of which included only one rare haplotype (Bat17) (Figs.2, S1). These two lineages corresponded to the two divergent haplogroups, Tsushima and Kuroshio, which were previously named for the two regional haplotype groups of Japanese *B. attramentaria*. These haplogroups occupy the north and south of Japan, corresponding, respectively to the routes of the Tsushima and Kuroshio Currents around the Japanese Islands (see figure1 of Kojima et al. 2004). Most of the haplotypes from Korea belonged to the Tsushima group, named for the haplotypes exclusively found in the north of Japan. In contrast, the rare haplotype Bat17, found in only three individuals in the southern part of Jeju Island, belonged to the Kuroshio group, the divergent lineage found mostly in the south of Japan. The haplotype network of Korean and Japanese *B. attramentaria* showed starbursts of two divergent haplogroups, Tsushima and Kuroshio (Fig.3A). Bat17 clusters with Kuroshio. Notably, most members of the Tsushima group were clustered around the dominant lineage, Bat1, one mutational step away, indicating that Bat1 represents a potential ancestral haplotype of this group, the descendants of which might have expanded in geography and demography (Fig.3B). Our IMa2 analysis of the pooled Korea–Japan data (No. of individuals of Kuroshio = 222 and Tsushima = 482) estimated the date of split between these divergent groups at approximately 400,000 years ago (ka) with a very wide confidence interval (95% highest posterior density interval (HPD): 170−2,500 ka) (Fig.3B). This type of uncertainty in our estimate may be the result of the short *COI* sequences (337 bp). IMa2 results for bidirectional migration rates between the groups showed an absence of gene flow. The effective population sizes of the Kuroshio and Tsushima groups were estimated to be approximately 130,000 (95% HPD of 70,000−230,000) and 120,000 (95% HPD of 60,000−200,000), respectively.

### Range shift and demographic expansion of the Korean *B**. **attramentaria*

**Figure 4:**
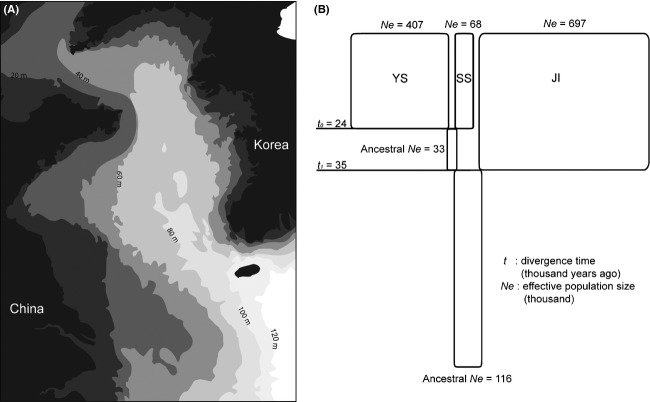
(A) Paleocoastlines at different depths in the Yellow Sea during the glacial and interglacial periods of the Quaternary. The darkest gray represents land at the present time. The lighter grays are areas submerged in water at different isobaths (Chun 2008). (B) Diverging processes of Korean *Batillaria attramentaria* are depicted under the Isolation with Migration (IM) model with the population demographic parameters of effective population sizes, splitting times, and migration rates between three geographical groups of the Yellow Sea (YS), the South Sea (SS), and Jeju Island (JI). The widths of rectangles proportionally represent hi-smooth means of effective population sizes of the present populations and hypothetical ancestral populations. The symbols of *t*_0_ and *t*_1_ correspond to the splitting times of ancestral populations into their two descendant populations, respectively. For simplicity, 95% HPD values of each parameter are not included, but can be seen in Figure S2.

A total of 197 *COI* sequences collected from thee different groups of Korean *B. attramentaria,* the Yellow Sea (YS), the South Sea (SS), and Jeju Island (JI), were analyzed under the Isolation with Migration model in IMa2. The run resulted in convergent marginal posterior density curves for six model parameters of effective population sizes, bidirectional migration rates, and divergence times for all three groups and their ancestral populations (Figs.4, S2). The density curves of the model parameters are shown in Figure S2 with narrow confidence intervals, except for divergence times. The estimates of effective population size for the YS, SS, and JI groups and their two ancestral population were approximately 407,000 (95% HPD: 110,000–3,760,000), 68,000 (95% HPD: 15,000–240,000), 697,000 (95% HPD: 200,000–3,700,000), 33,000 (95% HPD: 0–4,090,000), and 116,000 (95% HPD: 0–620,000), respectively (Fig. S2A). The divergence time parameter *t*_0_ for the split between the YS and SS groups was estimated at 24 ka, with a 95% HPD interval of 85–10 ka; *t*_1_ for the divergence between the JI and the ancestral population of the YS and SS was approximately 35 ka, with a 95% HPD interval of 105–22 ka (Figs.4B, S2c). In Figure S2C, the posterior density of the divergence time parameter *t*_0_ has a narrower interval than *t*_1_, with a long tail. The latter result showed that there was insufficient information in our *COI* data to specify the divergence time parameter. Our likelihood-ratio tests of nested models of gene flow, including comparisons between current populations and between ancestral populations, revealed that there was no statistically significant gene flow in any of them (Table3). In these tests, we did not examine populations with apparent peaks at a migration rate of zero (Fig. S2B).

**Table 3:** Tests of nested models of gene flow for three geographic groups of *Batillaria attramentaria* in Korea

Model (Θ)	Log ( 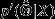 )	−2 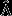	*P*	df
*m*_0>1_*m_0>2_ *m*_1>0_ *m*_1>2_ *m*_2>0_ *m*_2>1 _*m*_2>3_ *m*_3>2_	2.000	–	–	–
*m*_0>1_ = 0 *m*_0>2_ *m*_1>0_ *m*_1>2_ *m*_2>0_ *m*_2>1 _*m*_2>3_ *m*_3>2_	1.730	2.520	0.056	1
*m*_0>1_ *m*_0>2_ *m*_1>0_ *m*_1>2_ *m*_2>0_ = 0 *m*_2>1_ *m*_2>3_ *m*_3>2_	2.803	0.375	0.270	1
*m*_0>1_ *m*_0>2_ *m*_1>0_ *m*_1>2_ *m*_2>0_ *m*_2>1_ *m*_2>3_ = 0 *m*_3>2_	2.927	0.127	0.360	1
All equal migration rates	0.538	4.904	0.972	7
All zero migration rates	2.927	0.127	0.500	8

* The subscript numbers below migration parameter (*m*) denote identities of three different geographic populations and an ancestral population (0: YS, 1: SS, 2: JI, and 3: the ancestral population of YS and SS), and the abbreviations of populations are shown in Table1. The symbol “>“between two numbers indicates a direction of gene flow from a recipient population to a donor population in a time-forward manner, as described by Hey and Nielsen (2007). The column heads follow the definition given in detail in the same paper (Hey and Nielsen 2007).

### Reconstruction of the demographic history of *B**. **attramentaria* by Bayesian skyline plots (BSPs)

The summary statistics Tajima's *D* and Fu's *F*_*S*_ were calculated from each of the fifteen Korean *B. attramentaria* populations and for the four geographical groups separately (Table4). Three of four groups showed statistically significant negative values of Tajima's *D* and Fu's *F*_*S*_. In particular, Fu's *F*_*S*_ showed highly negative values: YS, −7.27; SS, −2.99; and NJI, −3.20. Among all 15 populations, only one, Gonam, showed significance in both Tajima's *D* and Fu's *F*_*S*_ (*P *<* *0.05). These summary statistics indicated that snail populations around the Korean peninsula might have experienced recent demographic expansions.

**Table 4:** Tajima's *D* and Fu's *F*_*S*_ test of selective neutrality

	Tajima's *D*		Fu's *F*_*S*_	
	*D*	*P*	*F*_*s*_	*P*
Yellow Sea (pooled)	−1.832	0.007*	−7.178	0.000*
1 Dobido	−1.055	0.176	−1.478	0.053
2 Gonam	−1.507	0.040*	−2.355	0.005*
3 Byunsan	0.000	1.000	0.000	–
4 Jindo	0.139	0.635	0.050	0.394
South Sea (pooled)	−1.566	0.036*	−2.989	0.015*
5 Yeosu	−0.594	0.267	−0.518	0.250
6 Geoje	−1.468	0.065	−1.402	0.021
7 Masan	0.850	0.832	2.010	0.804
North of Jeju (pooled)	−1.919	0.006*	−3.203	0.015*
8 Shinchang	0.000	1.000	0.000	–
9 Hyubje	−1.504	0.053	−1.680	0.016*
10 Hanlim	−1.141	0.053	−0.476	0.130
11 Sewha	0.826	0.821	3.754	0.955
12 Samyang	−1.055	0.213	−0.182	0.204
South of Jeju (pooled)	−0.5511	0.334	1.794	0.830
13 Shinyang	0.048	0.591	0.018	0.382
14 Kangjeong	−1.194	0.122	0.698	0.614
15 Pyosun	0.894	0.840	5.955	0.991

* *P *<* *0.05 for Tajima's *D* test, *P *<* *0.02 for Fu's *F*_*S*_ test.

**Figure 5:**
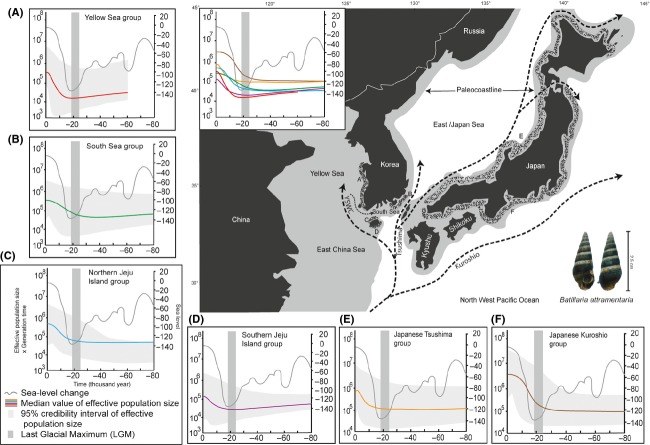
Upper right panel illustrates the map of paleocoastlines and ocean current systems around the Korean peninsula and the Japanese archipelagos during the glacial and interglacial periods of the Quaternary (Rella and Uchida 2014). The dark gray is the continental shelf; light gray is the territorial space that was above sea level when the sea level was 120 m lower than at the present day, and the patterned areas indicate six representative geographic groups of *Batillaria attramentaria*. Dashed lines with arrows represent prevailing regional currents of the Tsushima and Kuroshio and their branches. YSWC is an abbreviation of “Yellow Sea Warm Current”. Bayesian skyline plots (BSPs) illustrate the demographic histories of six discrete geographic groups of *B. attramentaria* from (A) the Yellow Sea, (B) the South Sea of Korea, (C) northern Jeju Island, (D) southern Jeju Island, (E) the Tsushima haplogroup of Japan, and (F) the Kuroshio haplogroup of Japan over the last 80,000 years in comparison with the global change in sea level (Siddall et al. 2003; Woodroffe and Webster 2014). For comparison, all of these BSPs are compiled in an inset on the map. Within each BSP panel, the median historical effective population size is represented by a colored line with the 95% confidence interval in gray. The historical sea-level change is shown by a gray line, and the period of the last glacial maximum (LGM: 26–19 ka) is marked with a vertical band in darker gray.

Given this result and the previous evidence of population growth of Japanese *B. attramentaria* (Kojima et al. 2004), we further examined the demographic history of this species around the coasts of Korea and Japan through the Bayesian skyline plot (BSP) method with particular focus on dating the changes in population size and comparing the degree and mode of these changes among representative geographic regions: four groups (YS, SS, NJI, and SJI) in Korea and two groups (Tsushima and Kuroshio) in Japan. As shown in Fig.5, all six groups showed consistent and exponential demographic increases since the last glacial maximum (LGM), approximately 26–19 ka. Although the 95% confidence intervals the BSPs were over one and half orders of magnitude and there was some variation in time and degree of the demographic expansions (see BSPs pooled in the inset of the map of Fig.5), the shape of BSPs across the six geographic groups was quite similar to each other, showing almost synchronous population expansions. It was also notable that the time of the expansions surprisingly coincided with the sharp rise in the sea level since the LGM (see the gray curve of global sea-level change in Fig.5). In addition, the BSPs commonly showed very similar flat periods with no change in population size after the LGM to 80 ka. Among the six current geographic groups, Kuroshio was the biggest, followed by Tsushima, and SJI was the smallest. These contrasts are approximately correlated with the size of areas sampled and geographic location. A comparison of the plots showed that Kuroshio began to increase slightly earlier and much more noticeably than the other groups. The present population is approximately 50 times greater than that before the LGM. The YS group was also dramatic in its increase in both rate and timing and showed the most recent start of population expansion, at approximately 15 ka (Fig.5A).

Our parallel analysis of mismatch distribution based on a sudden expansion model also supported the results of the BSPs. Mismatch distribution curves of all four geographic groups (YS, SS, NJI, and SJI) showed unimodal shapes for the expected distribution of pairwise sequence divergences under the assumption of a sudden demographic expansion model (Fig. S3A). In addition, the mismatch distribution of the four groups as a single pooled group indicated a demographic expansion (*P *=* *0.45) (Fig. S3E). The timing of the demographic expansions was estimated through the conversion of the resultant *tau* values (*τ*, population expansion time scaled by mutation rate in the range 0.8–2.4% per nucleotide per million years) from the analysis of the mismatch distribution. The beginning time of the sudden population expansion of each Korean *B. attramentaria* group was calculated as follows: YS (49–18 ka), SS (78–28 ka), NJI (140–50 ka), and SJI (39–14 ka), and for the whole Korean population, (86–31 ka).

## Discussion

Repeated glaciations during the Quaternary period resulted in substantial changes to the coastlines in East Asia due to its shallow and widespread continental shelves, particularly in the East China Sea and Yellow Sea. Accordingly, the historical change of the coastlines during the Quaternary period in East Asia casts questions on its role in the differentiation and speciation of marine organisms inhabiting coastal zones. Several previous phylogeographic studies on mollusks in this region including our study organism, the intertidal batillariid snail, *Batillaria attramentaria*, showed evidence of demographic growth (Kojima et al. 1997, 2000, 2004; Chiu et al. 2013; Xue et al. 2014) as a common effect of past climate changes on marine species, as in other oceans (Table1 and references therein, Grant et al. 2012). Generally, however, the detailed evolutionary and demographic consequences of the tremendous cyclic changes in the coastlines for marine species remain to be resolved beyond the broad trend of demographic growth. Indeed, there is a large gap in our knowledge about where, when, and to what degree the paleo-environmental changes affected the historical distribution and demography of marine species across their entire distributional ranges in East Asia.

In this study, we investigated in detail the evolutionary response of *B. attramentaria* to the coupled changes of climate and coastlines on a broader geographic scale than the previous study. The wide distribution of *B. attramentaria* along the coastlines of East Asia prompted us to predict that this species may contain highly resolved evolutionary information on the impacts of past climate changes followed by paleoceanographic changes on its evolution from local to regional scales and thus could be served as a model species for the speciation of gastropods in this region. To resolve the presumably coupled changes in the distribution and demography of *B. attramentaria*, as they have been commonly influenced by the concurrent environmental changes, we employed more realistic population genetic methods based on a coalescent framework: Isolation with Migration (IM) model for the divergence of multiple populations (Hey 2010a,b) and Bayesian skyline plot (BSP) method for the reconstruction of a detailed demographic history as applied to marine organisms (for review Ho and Shapiro 2011; Foote et al. 2013; Silva et al. 2014). As the result, these approaches enabled us to generate a more comprehensive and also more finely resolved understanding of the evolutionary response of *B. attramentaria* to paleo-environmental changes, highlighting a surprisingly strongly correlation between the mode of demographic expansion and climate change, particularly since the last glacial maximum (LGM: 26–19 ka) (Clark et al. 2009). The rising sea level and warming temperature since the LGM should have left pronounced genetic signatures of range shifts, geographic isolations, and demographic expansions in the genomes of living *B. attramentaria*, as discussed in detail below.

### Divergence of two allopatric haplogroups of *B**. **attramentaria* in East Asia

The molecular phylogeny of the genus *Batillaria* (Gastropoda: Batillariidae) in East Asia showed that there are four closely related species of snails around China, Japan, and Korea: *Batillaria attramentaria* (Sowerby, 1855), *B. flectosiphonata* Ozawa, 1996, *B. multiformis* (Lischke, 1869), and *B. zonalis* (Bruguière, 1792) (Kojima et al. 2001; Ozawa et al. 2009). The species statuses of them were turned out to be reliable given the highly supported monophyly of each species' individuals both in the tree of nuclear 28S rRNA and mitochondrial 16S rRNA (Ozawa et al. 2009) and the tree of mitochondrial *COI* (Kojima et al. 2001). Among the four species, *B. flectosiphonata* and *B. multiformis* are the most closely related to each other and *B. zonalis* takes the basal position. It is sure that *B. attramentaria* is robust in its species status.

Kojima et al. (2004) discovered two divergent allopatric haplogroups of *B. attramentaria*, “Tsushima” and “Kuroshio” in the north and south of the Japanese archipelago, respectively. The authors stressed that the geographic division might be the result of the warm Tsushima and Kuroshio Currents divergently flowing along the coasts of Japan (Fig.5). Increased sampling from the Korean coasts reveals that almost all individuals from Korea belong to the northern Tsushima haplogroups, except for three individuals from southern Jeju Island (SJI), which lies farthest south (Fig.4A). Overall, therefore, the distribution of the Tsushima haplogroup corresponds to the route of the warm Tsushima Current and its branches (Fig.3A). We therefore asked when and how these two haplogroup became separated.

The result of the Isolation with Migration (IM) model (Hey 2010b) suggests that the two haplogroups began to split from an ancestral population approximately 400 ka (Fig.3B). The divergence between the Tsushima and Kuroshio haplogroups cannot be accounted for without the bifurcating Kuroshio Current. The main Kuroshio Current splits into two discrete branches, forming the Tsushima and Kuroshio Currents, when it meets Kyushu Island. While the Tsushima Current extends directly to the East Sea/the Sea of Japan, the Kuroshio Current flows along the southern coast of Japan (Lie et al. 1998; Park et al. 2000) (see the map of Fig.5). Consequently, these bifurcating currents likely began to subdivide the *B. attramentaria* ancestral population into the two geographically distinct haplogroups approximately 400 ka; however, these data are not sufficient to explain the relatively deep divergence time because the rather continuous coastlines of the region might have allowed continuing or episodic contacts between the different lineages. As expected, some localities, particularly at the boundaries between the allopatric haplogroups, displayed mixed haplotypes: Yamada Bay, Maehama Tidelands, and Goto Island (Kojima et al. 2004). This type of mixture also exists in the SJI of Korea: the rare Kuroshio haplotype, Bat17, intermixes with the dominant Tsushima haplotypes (Figs.2, 3). Ongoing or recent long-distance gene flow seems highly unlikely because the genetically closest Kuroshio haplotype, “K13” (Kojima et al. 2004), which is one mutational step removed from Bat17, is distributed from Kushimoto to Yamada Bay, which is too far from Jeju Island to make dispersal likely (Fig.3A). Additionally, IMa2 analyses of migration rates among four geographic groups of Korean *B. attramentaria* showed no or nonsignificant gene flow among them, which rules out long-distance gene flow (Table3 and Fig. S2B). The second-closest Kuroshio haplotypes, “K20” and “K23,” which are two mutational steps removed from Bat17, occur in Maehama Tidelands and Goto Island in Japan. Interestingly, these two sites lie on the western side of Japan where the two haplogroups coexist. Geographically, Goto Island is closest to Jeju Island among all of the sites in Japan sampled by Kojima et al. (2004). The coexistence of the divergent haplogroups in the south of Jeju Island suggests the location and movement of an ancestral boundary between them. Because currently there is no allopatric Kuroshio haplogroup in Korea, we speculate that the rare Bat17 haplotype might be a historical remnant of the Kuroshio lineage that reached the SJI from nearby Japanese Islands, such as the closest Goto Island, possibly when the sea level was lower and the distance between the coasts of Korea and Japan was thus much smaller than in the present day (see the paleocoastlines in Fig.5). Alternatively, the southernmost population of the Tsushima haplogroup in Korea might have contacted the Kuroshio haplogroup historically, possibly in the south of Jeju Island, when the paleocoastline connected the island to the south (Fig.5). As marine transgression began during interglacial periods, such a contact might have disappeared, leaving only the southern side of Jeju Island as a zone of overlap of the two haplogroups.

From 400 ka onwards, there were four glacial cycles accompanied by four glacial maxima (see Fig.1, Siddall et al. 2003). During these maxima, the sea level dropped approximately 100 m, which might have caused the shallow continental shelf between the Korean peninsula and Japan to form a land bridge or extremely narrow canal, preventing the Tsushima Current from flowing into the East Sea/the Sea of Japan (Park et al. 2000; Kitamura et al. 2001). Thus, this land bridge or narrow channel-like seaway likely served as a barrier reinforcing the vicariance of *B. attramentaria* into the Tsushima and Kuroshio haplogroups. A similar pattern of disjunct distribution with a similar geographic boundary was observed in another gastropod species, the Japanese turban shell *Turbo cornutus*, which formed two divergent haplogroups that also exactly corresponded to the warm currents (Kojima et al. 1997). This concurrent vicariant pattern in different species strongly suggests that the common environmental conditions of the warm Tsushima and Kuroshio Currents and the paleoceanography of the Pleistocene period are responsible for the long-term distribution and geographic differentiation of marine species inhabiting shallow coastal areas around Japan.

The initial subdivision of *B. attramentaria* might have occurred through a series of colonizing events in the East Sea/Sea of Japan by founders from the southern region of the Japanese archipelago. This scenario is indirectly supported by the evidence of migration of warm-water planktonic foraminifera and mollusks that were under the same effects of the Tsushima Current (Kitamura et al. 2001). In fact, *B. attramentaria* and the other batillariids in East Asia have a southern origin (Ozawa et al. 2009). Fossil records and the molecular phylogeny of the family Batillariidae suggest that the recent genus *Batillaria* living in East Asia was derived from ancestors present in Australasia and that the Oriental *Batillaria* began to appear in the Early Miocene (Ozawa et al. 2009). Apart from this deep origin of the Oriental batillariids, our estimation of effective population size of *B. attramentaria* from the present to the recent past of 80 ka also supports the hypothesis of a southern ancestor because the southern “Kuroshio” population has long been larger than the northern “Tsushima” population (Fig5E, 5F). Therefore, parsimonious reconstruction of the vicariance of *B. attramentaria* suggests that the initial colonization of the East Sea/Sea of Japan by the southern ancestor of *B. attramentaria* and subsequent genetic differentiation has long been reinforced not only by divergently flowing ocean currents but also by the end of the connection of the East Sea/Sea of Japan to the southern neighboring sea via the Tsushima Strait that was present during glacial maxima (e.g., paleocoastlines in Fig.5).

### Marine transgression of the Yellow Sea and its implications for the geographical differentiation of *B**. **attramentaria*

Four differentiated geographic groups exhibiting significant pairwise *F*_*ST*_ values relative to each other were found along the coasts of Korea: the Yellow Sea (YS), the South Sea of Korea (SS), and the northern (N) and southern (S) areas of Jeju Island (JI) (Fig.2A and Table2). These geographic groups are mostly accounted for by the different coasts around the Korean peninsula and the Island. These coasts were not always in their current positions. The level of the Yellow Sea with low gradients was lower on average by 120 m and so the shelf of the sea experienced subaerial exposure during the LGM (Shinn et al. 2007). As illustrated in Fig.4A, the sea level has risen rapidly since the LGM. Consequently, the shoreline configuration and oceanographic regime changed significantly as the coastline moved inland, corresponding to the rate of sea-level change (a series of rapid rises interrupted by long-term, slow rises) (Liu et al. 2004). As mentioned earlier, *B. attramentaria* is exclusively confined to the narrow intertidal zone and has a reproductive stage of directly developing larvae (Adachi and Wada 1999). Thus, *B. attramentaria* should have been subjected to the paleoceanographic shifts in coastlines in response to climate change.

Therefore, we attempted to understand the genetic differentiation of Korean *B. attramentaria* as a series of population divergences from an ancestral population into the current geographical groups along the shifting coastlines over time. To this end, the Isolation with Migration (IM) model for more than three populations (Hey 2010a,b) was applied to the subsample of Korean *B. attramentaria* such that the drastic event of postglacial marine transgression in the Yellow Sea was reflected in intraspecific divergences as, for example (YS, SS), JI) (Fig.4). Because the transgression must have accompanied isolations and range shifts of ancestral *B. attramentaria* populations as the paleocoastlines shifted northwards from the south during interglacial periods (Figs.4A, 5), we assumed that the first divergence event between the JI group and the ancestor of the YS and SS groups was followed by the second one, resulting eventually in the current YS and SS. In this context, the intriguing question was when and how strongly the past coastline shifts left their marks on the current genetic variation in Korean *B. attramentaria* during the Late Pleistocene period.

The IMa2 analysis based on the reconstructed divergences enabled us to estimate the population demographic parameters of the consecutive divergences from the population *COI* sequences: effective population sizes, migration rates, and divergence times of the first and second divergences (Figs.4B, S2). The two consecutive divergence times (*t*_0_ and *t*_1_) were estimated as 24 ka (95% HPD: 85–10 ka) and 35 ka (95% HPD: 105–22 ka), respectively. In overall, the estimated duration of the divergences of the three geographic groups and the subsequent demographic increases of each group overlaps with the period of marine transgression in the Yellow Sea from the LGM onwards. The split time (*t*_0_) was not significantly difference from that of the prior split (*t*_1_), suggesting that the two divergences may reflect a process of very successive isolations among the three geographic groups (YJ, SS, and JI) from the LGM onwards (Fig. S2C). Overall, our results demonstrate that the present genetic variation in Korean *B. attramentaria* could be traced to the initial splitting event of the most recent common ancestral population, approximately 35 ka and thus could correspond to the phylogeographic and demographic events occurred up to this time.

Importantly, the IMa2 analysis showed remarkable increases in the effective population size of the YS and JI groups relative to their ancestral populations, although the effect was weak in the SS group (Fig.4B). Compared to the effective population size of the most recent common ancestors (116 thousand), the sum of all of the current effective population sizes (1,172 thousand) is ten times greater. The duration of 35 ka for the demographic increase attracts our interest because it includes the LGM and thus a series of events from that time: the last marine transgression around the Korean peninsula, which accompanied tremendous shifts in coastlines, and the isolation of Jeju Island from the terrestrial part of the peninsula (Fig.4A). Therefore, the demographic growth of Korean *B. attramentaria* should have occurred while the shifts of coastlines were resulting in expansion of habitats and the air temperature increased by approximately 8–13°C, reaching the present levels (Park and Yi 1995). The new habitats in the Yellow Sea must have been colonized by founders conveyed through the northward wave of range expansion, and the early founders would have sharply increased in number as the thermal environment became favorable to the snails. This evidence of an exponential increase in the effective population size of *B. attramentaria* around Korea within the last thirty-five thousand years was corroborated by BSP analyses in detail (Fig.5).

An additional interesting point related to the IMa2 results is that migration rates between any pair of geographic groups were zero or negligible (Table3 and Fig. S2). Zero or nonsignificant gene flow is consistent with the reproductive mode of this species, which has a directly developing larval stage (Adachi and Wada 1999), and also with the significant differentiation among Japanese *B. attramentaria* populations (see Table 5, Kojima et al. 2004). Therefore, each local group may tend to retain population genetic variation much more faithfully, corresponding to respective historical environmental changes without being affected by geographically distant groups through gene flow. We consider that the lack of gene flow is a universal feature of *B. attramentaria* that holds across almost the entire distributional range. This feature, in turn, justifies our handling of local populations for the present IMa2 and BSP analyses by sorting and pooling them into a series of geographic groups according to genetic and geographic closeness, which might ultimately reflect natural units of populations sharing demographic and distributional histories apart from each other within certain time frames.

### Postglacial demographic expansion of *B**. **attramentaria*

As suggested by the IMa2 results, our Bayesian skyline plot (BSP) analyses detected historical demographic expansions of *B. attramentaria* across its distributional range since the LGM (26–19 ka) (Fig.5). We applied the BSP method to six geographical groups that are both genetically and geographically distinct. The BSPs of the six groups consistently showed very similar curves of recent exponential demographic growth in both time and mode, although there was slight variation. To our surprise, the demographic expansions are highly correlated with the curve of global sea-level change (see gray curve in Fig.5). In other words, the demographic change in *B. attramentaria* appears to be almost synchronized with the climate change from the LGM and its transition to the present day. Note that the period of LGM (26–19 ka) is near the start of the increases in the demographic curves (Fig.5), suggesting its correlation with the demographic expansions.

The widespread demographic expansion could be explained by the transition between contrasting environments of the LGM and after it. The global sea-level and temperature rise driven by climate changes since the LGM should have brought sudden changes in the physical and thermal environment in the seas surrounding Korea and Japan. The most important environmental changes include the expansion of habitats following shifts in coastlines during the interglacial marine transgression and mild temperatures in the region. These environmental changes must have been so powerful in both scale and degree that the demographic growth of *B. attramentaria* occurred almost simultaneously throughout the entire distributional range of this species. Despite this wide and concurrent expansion, there was a slight difference in the time of initiation of the expansion among locations. For instance, the Kuroshio group began to increase approximately 35 ka, but the Tsushima group began at approximately 19 ka. The Kuroshio group of *B. attramentaria* was estimated to have increased about to fifty times the size it was before the LGM and, as a result, is five times larger than the Tsushima group (Fig5E, F). This difference could be related to a time gap between the warming effects of the two different ocean currents around Japan; while the warm Kuroshio Current easily carried heat to the southern coastal region of Japan, the warm Tsushima Current could not deliver heat to the East Sea/Sea of Japan due to the land bridge between Korea and Japan in the early interglacial period (Fig.5); however, as sea level gradually increased, the channel-like seaway, Tsushima Strait, widened, and the warm Tsushima Current flowed into the East Sea/Sea of Japan, creating thermally favorable environments to the Tsushima group later than in the Kuroshio group.

Unfortunately, there has been no study directly testing the effect of water temperature for the survival and reproduction of *B. attramentaria*. The only literature from which we could gain insight into this question was a field study on the growth and longevity of *B. attramentaria* inadvertently introduced to the western coast of North America from Japan with the Japanese oyster *Crassostrea gigas* (Yamada 1982). Yamada (1982) reported that warm winter temperatures were linked to good recruitment of juveniles the next spring in British Columbia, while unusually cold winter temperatures were linked to poor or late recruitment over 10 years of observation. This short-term field observation is consistent with our argument that long-term warm-water temperatures might be favorable to the survival and reproduction of *B. attramentaria,* leading to its demographic expansion since the LGM.

The extent of demographic expansion, over ten times, is also remarkable in the Yellow Sea (YS) group with its relatively late initiation (approximately 15 ka). The estimates of degree and time are similar to those from IMa2 (about twelve times and 24 ka). The slight difference in time estimation between the methods may be due to differences in the exact meaning of the time. That is, the value of 24 ka is split between the YS and SS, rather than reflecting the true starting time of the demographic increase in the YS. As mentioned above, the demographic history of the YS group may illustrate the response of *B. attramentaria* to habitat range and thermal condition following the last marine transgression since the LGM. The effect of range expansion on demographic expansion was also evident in the other batillariids living around New Zealand, *Zeacumantus subcarinatus* and *Z. lutulentus* (Keeney et al. 2013). These sister snail species also showed sudden demographic increases, but the pattern was apparent only in the area between the islands where marine transgression was evident during the interglacial periods. Therefore, the degree of demographic expansion in the YS could be attributable to the amplifying effect of the range expansion and warming temperature.

Notably, the immediate and faithful demographic response of *B. attramentaria* to the most recent climate change since the LGM is exceptional. The BSPs reconstructed from the *COI* sequences clearly demonstrate that most of the variation was shaped during the demographic expansion since the LGM, but there are no further informative coalescent events in *COI* lineages beyond 35 ka, making it impossible to estimate effective population size. It is likely that older variation might have been erased due to severe bottlenecks during glacial periods before the LGM. This speculation is based in the immediate demographic response of *B. attramentaria* even to harsh environments. Finally, in relation to the time window of the BSP method, a single locus of mitochondrial DNA may be not sufficient to detect old coalescent events because the effective population size of mitochondrial loci is smaller than that of nuclear genetic loci. Thus, DNA sequence data of multiple nuclear loci will be invaluable to clarify the intriguing question on the role of the precedent glacial maxima before the LGM.

As explained earlier, historical population growths associated with the past climate during the Late Pleistocene changes were demonstrated in several phylogeographical studies of marine fish and mollusks in East Asia (Kojima et al. 1997, 2000, 2004; Liu et al. 2006; Han et al. 2008; Kwan et al. 2012; Chiu et al. 2013; Xue et al. 2014). Taken together with the previous studies on the marine gastropods in East Asia, the Japanese turban shell *Turbo cornutus* (Kojima et al. 1997, 2000), the moon turban snail *Lunella granulata* (Chiu et al. 2013), and the intertidal snail *Batillaria attramentaria* (Kojima et al. 2004), our present study demonstrates the importance of the climate changes since the Late Pleistocene and the diversifying effect of the regional and ocean currents to both the demography and genetic differentiation of gastropods in this region. Therefore, the present and previous results from the three gastropod species suggest that the historical location of regional geographic barriers generated by lands and divergent oceanic currents and the range shift of marine habitats triggered by sea-level changes, particularly in the YS, should be considered for the evolution and distribution of marine organisms in East Asia.

## Conclusions

The molecular population data from the *COI* sequences of the marine batillariid gastropod, *B. attramentaria*, throughout the coasts of Korea and Japan provided unprecedentedly detailed information about the distributional and demographic history of its populations. Our results indicate that the repeated climate and oceanographic changes in East Asia during the Late Pleistocene have played an important role in the phylogeography and demography of this species, leaving evidence of the following population genetic processes in the *COI* sequences of *B. attramentaria* on various temporal and spatial scales. Divergence population genetic inference from the data demonstrated that (1) the two deep lineages of the geographically separated groups, “Kuroshio” and “Tsushima,” of this species began to diverge approximately 400 ka and then have occupied geographically separated subregions in the north and south, (2) the geographic differentiation of Korean *B. attramentaria* could be explained by continuing divergences of an southern ancestral population into Jeju Island, the Yellow Sea, and the South Sea of Korea, (3) the estimates of population demographic parameters for these processes are remarkably consistent with the oceanographic history of marine transgression around the Korean peninsula after the LGM, and finally, (4) the results of range shifts and demographic growths and the timing of the each event were largely consistent with those of BSPs. Finally, the Bayesian skyline plot (BSP) analyses revealed that the historical effective population sizes of local groups of *B. attramentaria* have drastically increased since the last glacial maximum (LGM: 26–19 ka). The relative concurrence and similarity in the modes of the demographic expansions at six representative geographic groups around Korea and Japan significantly coincided with the postglacial rise in sea level and seawater temperature in the region. Therefore, such widespread demographic expansions must have started as interglacial marine transgressions and warming temperatures generated of new habitats and more favorable water environments for *B. attramentaria* since the LGM. The remarkable association between the genetic variation of *B. attramentaria* and past environmental changes, particularly from the LGM onwards, could be attributable to the key features of this species: Habitats confined to narrow intertidal zones and a reproductive stage of directly developing larvae that limits dispersal capability. To our knowledge, *B. attramentaria* is the first marine species in the northwest Pacific for which historical distribution and demography have been resolved in detail over space and time, and its recent demographic expansion has been found to be directly associated with the coupled changes of coastlines and warming seawater temperatures since the LGM. Therefore, the current results could be used to generate working hypotheses for future phylogeographic and demographic studies of other marine species in the region.

## Supporting Information

Figure S1. Neighbor-joining tree of twenty unique haplotypes of *COI* (619 bp) from Korean *Batillaria attramentaria*. *Batillaria multiformis* was used as an outgroup.

Figure S2. The marginal posterior-probability distributions for population-demographic parameters of (A) population sizes, (B) migration rates, and (C) divergence times of IMa2 analyses with three populations of today: Yellow Sea (YS) group, South Sea (SS) group, and Jeju Island (JI) group, as described in figure 3..

Figure S3. Observed and expected distribution of pairwise sequence divergences under a sudden expansion model (Rogers and Harpending 1992) in (A) the Yellow Sea group, (B) the South Sea group, (C) the group of northern Jeju Island, and (D) the group of southern Jeju Island, (E) the pooled four groups of Korean *B. attramentaria*, (F) the pooled of Kuroshio group and Tsushima group of Japanese *B. attramentaria*. Horizontal axes represent the number of pairwise differences in nucleotide sequences and vertical axes do the number of occurrence. The comparative two lines represent the distributions: one of expected under the model of recent demographic expansion and the other observed from data.
