# Supplementary material for: Postglacial range shift and demographic expansion of the marine intertidal snail Batillaria attramentaria
Source: Ecol Evol. 2014 Dec 28;5(2):419–35. doi: 10.1002/ece3.1374 (PMC4314273; doi:10.1002/ece3.1374)
Supplement: Supplementary file 1 — Figure S1. Neighbor-joining tree of twenty unique haplotypes of COI (619 bp) from Korean Batillaria attramentaria. Batillaria multiformis was used as an outgroup. [file ece30005-0419-sd1.docx]

**Evolution and Ecology**

**Supporting information**

# Post-glacial range shift and demographic expansion of the marine intertidal snail *Batillaria attramentaria*

Phuong-Thao Ho^1^, Ye-Seul Kwan^2^, Boa Kim^2^, Yong-Jin Won^1, 2, 3, †^

^1^Division of EcoCreative, ^2^Division of EcoScience, ^3^Department of Life Science, Ewha Womans University, 52 Ewhayeodae-gil, Seodaemun-gu, Seoul 120-750, Korea

†Corresponding author e-mail: won@ewha.ac.kr

Tel: +82-2-3277-4471

Fax: +82-2-3277-2385


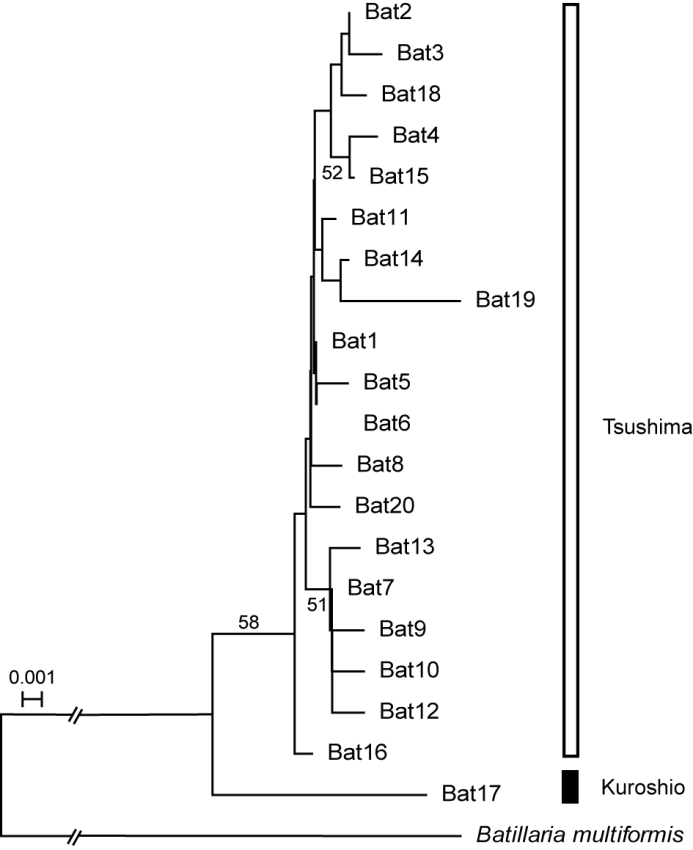


Firgure S1: Neighbor-joining tree of twenty unique haplotypes of *COI* (619 bp) from Korean *Batillaria attramentaria*. *Batillaria multiformis* was used as an outgroup.


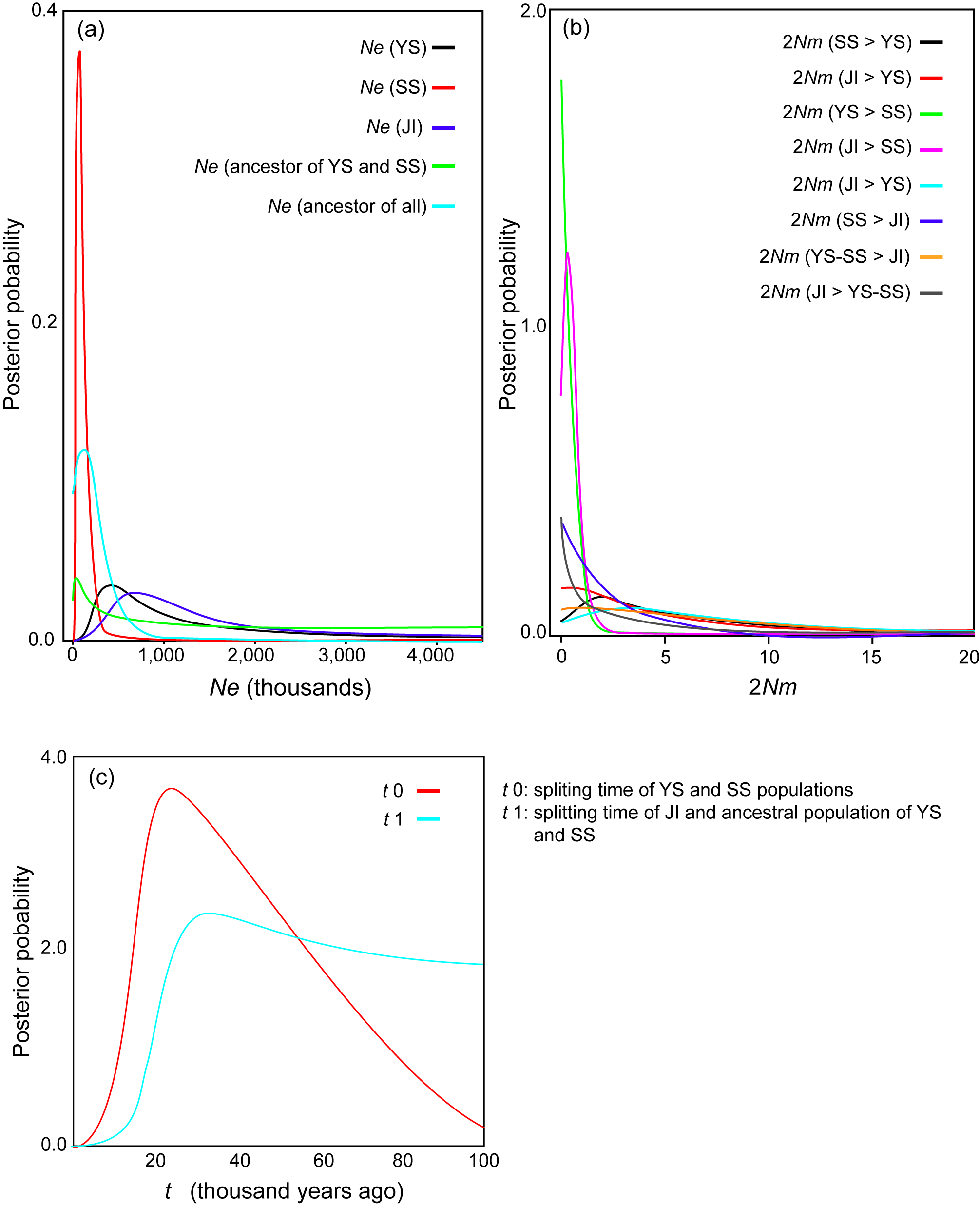


**Figure S2**: The marginal posterior-probability distributions for population-demographic parameters of (a) population sizes, (b) migration rates, and (c) divergence times of IMa2 analyses with three populations of today: Yellow Sea (YS) group, South Sea (SS) group, and Jeju Island (JI) group, as described in Figure 3. The coloured curves are shown with each corresponding legend in the panel. (b) The direction of gene flow is indicated by “>” between the donor and recipient populations, in that order. For example, “SS > YS” indicates gene flow from SS to YS, going forward in time. The symbol SS-YS in (b) indicates an ancestral population of SS and YS.
