# Supplementary figures and images for: Postglacial range shift and demographic expansion of the marine intertidal snail Batillaria attramentaria
Source: Ecol Evol. 2014 Dec 28;5(2):419–35. doi: 10.1002/ece3.1374 (PMC4314273; doi:10.1002/ece3.1374)

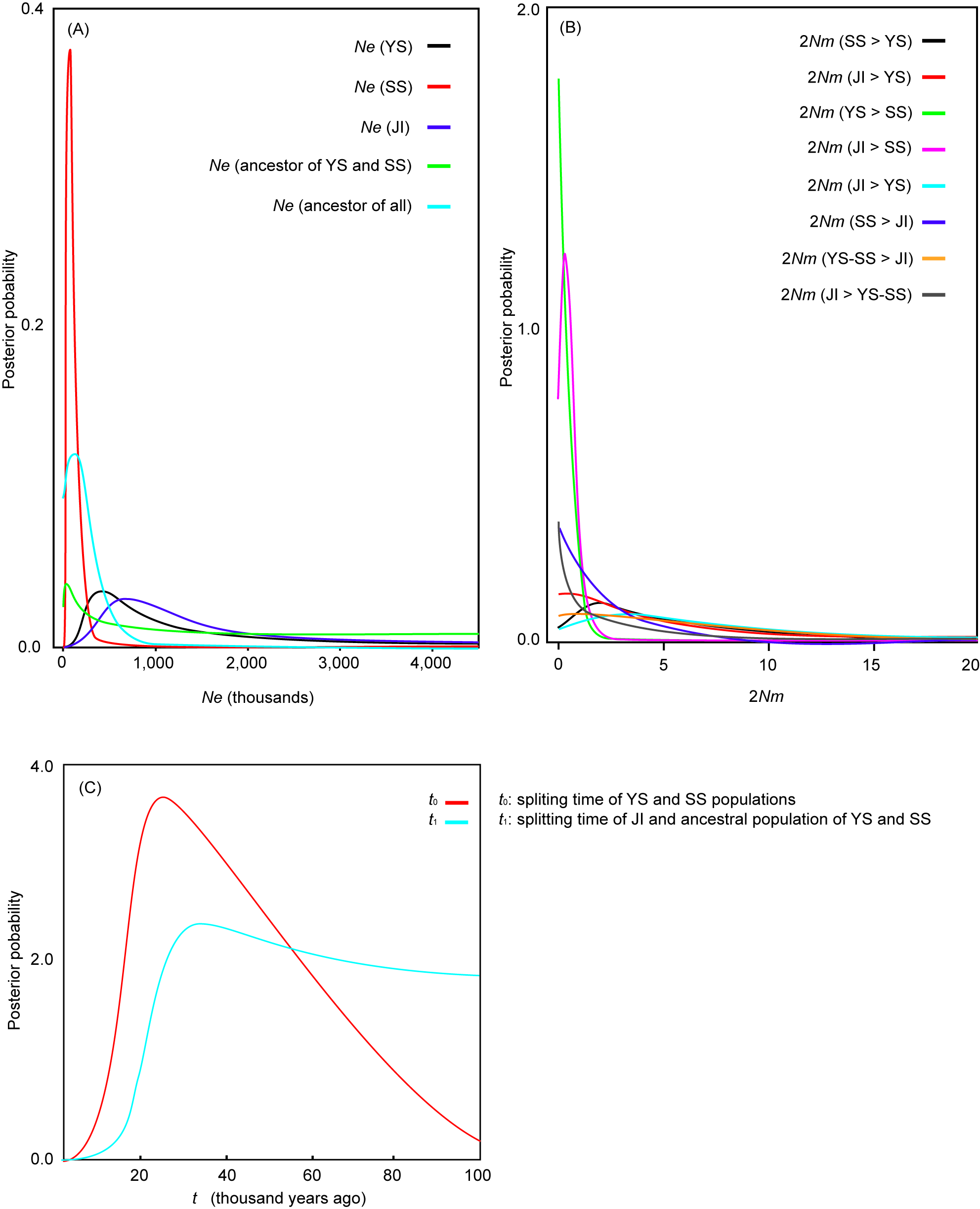

Supplement: Supplementary file 2 — Figure S2. The marginal posterior-probability distributions for population-demographic parameters of (A) population sizes, (B) migration rates, and (C) divergence times of IMa2 analyses with three populations of today: Yellow Sea (YS) group, South Sea (SS) group, and Jeju Island (JI) group, as described in figure 3.. [file ece30005-0419-sd2.tif]

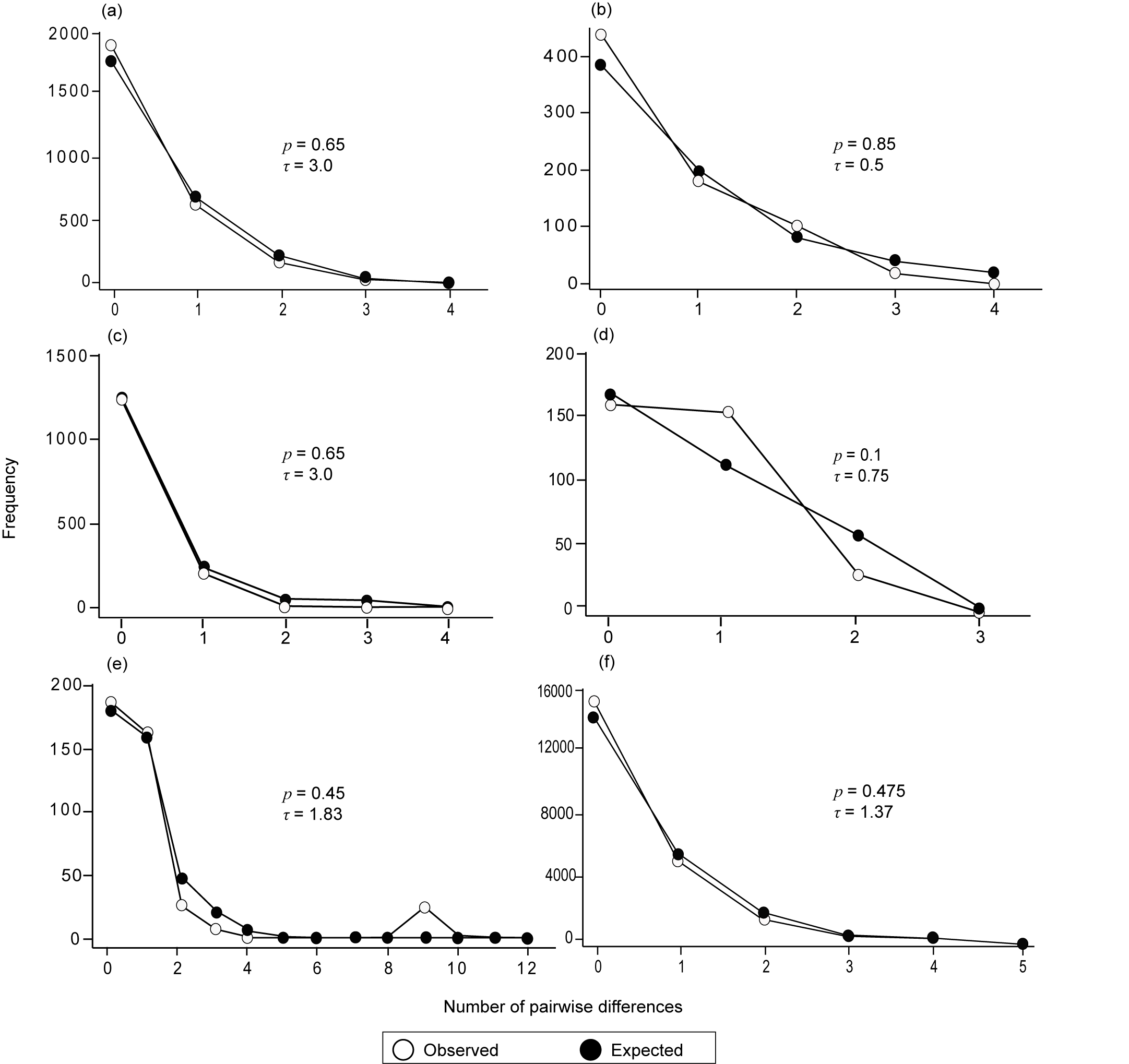

Supplement: Supplementary file 3 — Figure S3. Observed and expected distribution of pairwise sequence divergences under a sudden expansion model (Rogers and Harpending 1992) in (A) the Yellow Sea group, (B) the South Sea group, (C) the group of northern Jeju Island, and (D) the group of southern Jeju Island, (E) the pooled four groups of Korean B. attramentaria, (F) the pooled of Kuroshio group and Tsushima group of Japanese B. attramentaria. Horizontal axes represent the number of pairwise differences in nucleotide sequences and vertical axes do the number of occurrence. The comparative two lines represent the distributions: one of expected under the model of recent demographic expansion and the other observed from data. [file ece30005-0419-sd3.tif]
